# Skills development in infants: a possible role for widespread neurogenesis?

**DOI:** 10.3389/fnbeh.2013.00178

**Published:** 2013-12-02

**Authors:** Dekel Taliaz

**Affiliations:** Department of Neuroscience and Mental Health, The Hospital for Sick ChildrenToronto, ON, Canada

**Keywords:** neurogenesis, skills, child development, sensitive periods, postnatal, infant, cortex

There are differences in the way infants learn, perceive, and understand the environment as compared to adults (Bornstein, 1989; Nelson, 2000; Craik and Bialystok, 2006). While an adult has an established, clear perception of his environment, infants are still forming perceptions of the world (Hall et al., 1989; Holt, 1991; Zitelli and Davis, 2007). The mechanisms underlying these differences are not fully understood, but studies analyzing structural changes in infant brains give credence to a few suggestions, including increased synaptogenesis, dendritic arborization, myelination, and potentially even widespread neurogenesis.

## Skills development and sensitive periods

**Figure 1:**
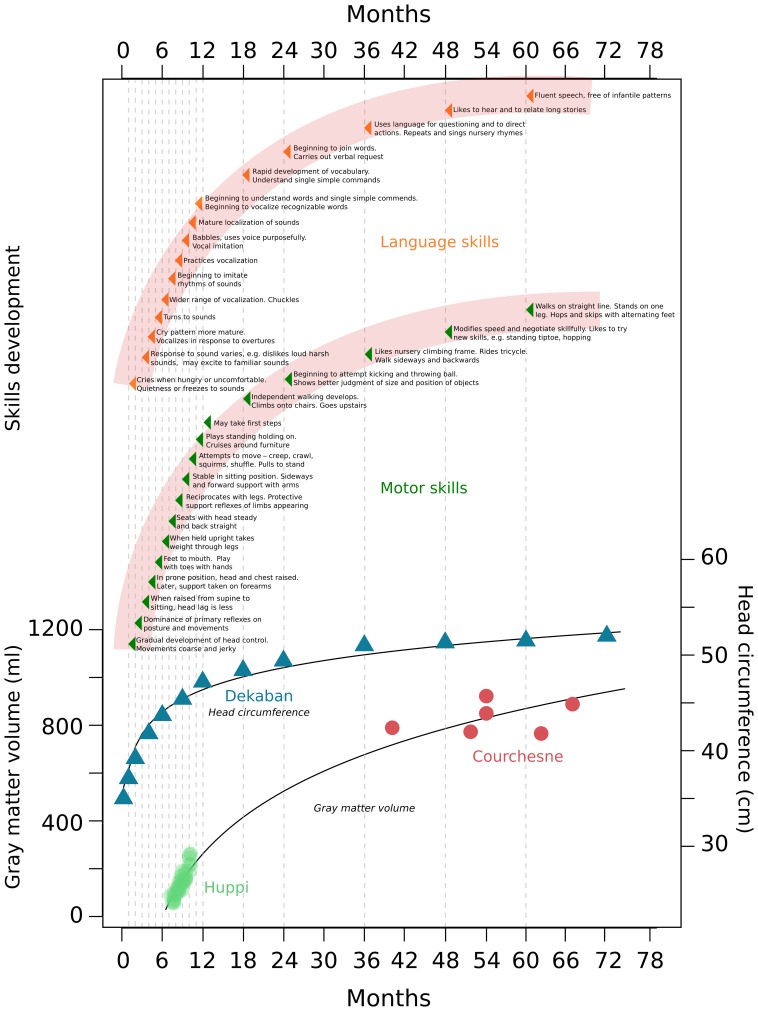
**Gray matter, head circumference, and skills development during the first years of human life**. This figure combines data from different studies measuring gray matter and head circumference increases, with the normal development of human skills as described in child development text books. Data was taken from Dekaban, 1977; Huppi et al., 1998; Courchesne et al., 2000 and Holt, 1991.

Early experience plays an important role in rapid cognitive development in infancy (Nelson, 2000). During the first year of life, newborns transform from totally dependent, wholly reflexive neonates to infants who recognize, react, and control their environment (Hall et al., 1989; Holt, 1991) (Figure 1). At this stage, infants establish bases for numerous skills and activities that allow for individual existence (Figure 1). Within the next 4 years (ages 1–5) skills rapidly develop; motor skills, such as running, hopping, climbing, throwing and catching emerge, as well as the ability to recognize similarities and differences between objects (Hall et al., 1989; Holt, 1991) (Figure 1). Human and animal studies demonstrated periods during development—termed *sensitive periods*—during which particular experiences exert an extraordinary and dramatic influence over brain structure and function (Nelson, 2000; Craik and Bialystok, 2006). For example, exposure to language in the first year of life has a profound effect on how one will later come to discriminate and recognize the sounds of that language; even as early as 6 months of age the ability to discriminate phonemes from languages to which infants are not exposed greatly diminishes (Kuhl et al., 1992). The acquisition of language continues to develop rapidly and is maintained into older adulthood, and a lack of exposure to speech or sign early in development results in the lack of normal language later in life (Fromkin et al., 1974; Flege et al., 1999; Mayberry et al., 2002; Mayberry and Lock, 2003; Kuhl et al., 2005). Similarly, visual experience in early life is necessary for the development of stereoscopic vision, and different sensitive periods for different aspects of vision exist in human development (Lewis and Maurer, 2005, 2009). In cats, depriving vision to one eye at a specific time point in infancy compromises stereoscopic vision in life, and affects the normal and complete elaboration of cell columns within the visual cortex corresponding to the deprived eye (Crair et al., 1998). Sensitive periods also exist for higher cognitive processes. Exposure to a healthy caretaking environment in the early years of human life was shown to be necessary for normal emotional development (Nelson, 2000), and deprivation studies of adopted children demonstrate that cognitive and emotional deficits can be ameliorated if children are adopted before their first birthday (Benoit et al., 1996; Nelson, 2000). Sensitive periods during development provide organisms a unique opportunity to adapt to the specific environments to which they are exposed. It is logical to assume that unique structural and functional changes in the brain underlie these sensitive periods.

## Brain development during early years of life

Between 7 days and 20 years old, humans exhibit a 1.5 fold increase in head circumference, with a 1.45 fold increase occurring between the ages of 7 days and 6 years (Dekaban, 1977) (Figure 1). Magnetic resonance imaging studies show a large increase in brain volume in the first years of human life. From 29 to 41 weeks post-conception, total brain tissue volume increases linearly at a rate of 22 ml/wk (Huppi et al., 1998). Total gray matter shows a linear increase in relative intracranial volume of approximately 1.4% per week, and pronounced increases in total gray matter reflect primarily a 4-fold increase in cortical gray matter (Huppi et al., 1998) (Figure 1). From early (26 months) to later (6–9 years) childhood, gray matter increases by 13% (Courchesne et al., 2000) (Figure 1). Robust changes in myelinated white matter also occur. Between 29 and 34 weeks post-conception the relative intracranial volume of myelinated white matter is as low as 1–2% of total intracranial volume (Huppi et al., 1998). Then, between 36 and 41 weeks post-conception a fivefold increase of myelinated white matter occurs to about 5% of total intracranial volume (Huppi et al., 1998). Later, between 19 and 33 months to 12–15 years white matter increases by 74% (Courchesne et al., 2000). These robust changes in gray and white matter are suggested to be mediated by profound changes in synaptogenesis, dendritic arborization, and myelination; in turn, these processes are suggested to play a role in the development of human skills and creation of sensitive periods (Brown, 1987; Bornstein, 1989; Crair et al., 1998; Craik and Bialystok, 2006). Widespread postnatal neurogenesis in infants may also have a role in these changes, but it has generally not been considered by researchers studying human skills development.

## Structural changes parallel skills development and sensitive periods

Changes in myelinated white matter, largely observed in infancy, were suggested to respond to experiences in a manner that affects neuron function, thereby affecting information processing and performance (Nelson, 2000; Fields, 2010). In a similar way, gray matter changes in early life, which are believed to be a result of synaptogenesis and dendritic arborization, seem to be involved in these developmental changes (Craik and Bialystok, 2006; Fields, 2010). Indeed, changes in myelination, synapse formation, and dendritic arborization were shown to underlie learning and memory throughout life. For example, increased myelination correlates with the number of hours a professional musician practices (Ullen, 2009), and adult subjects show increased white matter structural organization in a brain region important for visuo-motor control 6 week after learning to juggle (Scholz et al., 2009). Experience-dependent reorganization of the human motor cortex, which may underlie the acquisition and retention of motor skills, was also observed (Karni et al., 1995), and specific synaptogenesis and dendritic arborization of the forelimb motor cortex was shown to occur in response to the development of skilled forelimb movements in rats (Greenough et al., 1985; Withers and Greenough, 1989; Black et al., 1990; Kleim et al., 1997, 2002). In infancy humans experience skill development that is faster and more robust than any other period in life (Nelson, 2000; Craik and Bialystok, 2006). Skill development in infants, and by association the related sensitive periods, require particular and distinct mechanisms that may only occur at specific time points. The robust and wide spread increase of gray and white matter during infancy suggests that synaptogenesis, dendritic arborization and myelination occur at higher rates during these periods (Nelson, 2000; Craik and Bialystok, 2006). Importantly, another mechanism that may be distinct to infants is widespread neurogenesis. Adding new neurons can augment the creation of dendritic arborization and new synapses. Furthermore, postnatal neurogenesis may be one cause of delayed postnatal myelination, as a delay would be necessary to allow for the incorporation of new neurons throughout the brain. Considering the now established involvement of adult neurogenesis in learning and memory (e.g., Breton-Provencher et al., 2009; Lazarini et al., 2009; Valley et al., 2009; Sultan et al., 2010; Alonso et al., 2012) this potential mechanism for learning in early life must be taken seriously. At the same time, we must recognize that learning and memory mechanisms during development are probably different than in adults. For example, while a significant increase of gray and white matter occurs during infancy, only microscopic changes in gray and white matter were shown to be sufficient for learning and memory in adults (Draganski et al., 2004; Scholz et al., 2009). In the same manner, while infant neurogenesis may partially underlie the significant gray matter increase during infancy, adult neurogenesis in the rodent olfactory bulb was shown not to change the volume of the neurogenic region (Richard et al., 2010).

To date, few researchers have considered widespread neurogenesis as a potential mechanism because it was generally accepted that postnatal neurogenesis did not occur (Rakic and Sidman, 1968; Rakic, 1985). However, the available data to date is not conclusive, which leaves open a major question in the field of developmental neurobiology.

## Postnatal neurogenesis within the brain

In 1962–1966 Altman and Das published a series of studies that overturned the long-held dogma that the adult mammalian brain has no capacity for generating new neurons (Altman, 1962, 1963; Altman and Das, 1965, 1966). These studies showed that new neurons are formed within the dentate gyrus of the hippocampus and subventricular zone (SVZ) of infant and adult rats. Moreover, these studies have described the formation of new neurons specifically during infancy within additional brain regions, including the neocortex, thalamus and medulla. However, these results were met with great skepticism, mainly due to studies which reported that all neurons of the non-human primate brain are generated prenatally, except for some granule cells of the cerebellum and the hippocampus which continue their genesis for several months after birth (e.g., Rakic and Sidman, 1968; Rakic, 1985). Nevertheless, new studies using transgenic mice and novel tracing methods demonstrate massive migration of new-born neurons from the SVZ into numerous forebrain regions, including the cortex, striatum, and nucleus accumbens, mostly during infancy (De Marchis et al., 2004; Inta et al., 2008). Moreover, it is now established that the adult human and non-human primates brain retains the capacity to generate new neurons in the subgranular zone of the dentate gyrus (SGZ) (Eriksson et al., 1998; Kornack and Rakic, 1999; Aizawa et al., 2009). In addition, SVZ astrocytes were identified as neural stem cells in the adult human brain (Sanai et al., 2004), and the pathway through which newly born SVZ cells migrate and become mature neurons in the human olfactory bulb was identified (Curtis et al., 2007).

To this date, it is still unclear whether neurogenesis occurs in the neocortex and subcortical regions of the infant human brain. Some studies have not found any evidence for postnatal neurogenesis. These studies, which took advantage of the genomic integration of ^14^C (generated by nuclear bomb tests during the Cold War) to establish the age of neurons did not find evidence for postnatal neurogenesis in the major areas of the human cerebral neocortex (Spalding et al., 2005; Bhardwaj et al., 2006). However, in these studies the researchers used the neuronal nuclei (NeuN) marker in order to sort the cells for the ^14^C measurements. This fact is important because even though the expression of NeuN is observed in most neuronal cell types throughout the nervous system of adult mice, some major cell types appear devoid of immunoreactivity (Mullen et al., 1992). Therefore, it is possible that postnatal neurogenesis was not detected at the ^14^C studies due to the fact that these neurons (at least in measured regions) do not express NeuN. Indeed, a very recent study which has used the immature neuronal markers doublecortin and b-III tubulin described a major period of neurogenesis and neuronal migration from the SVZ to the human ventromedial prefrontal cortex (Sanai et al., 2011). Neurogenesis was shown to extend well into postnatal life, but to be largely limited to early childhood (around 18 months) (Sanai et al., 2011). More specifically, the infant human SVZ contains an extensive corridor of migrating immature neurons before 18 months of age, but this germinal activity subsides in older children (3–7) and is nearly extinct in adults (30–84) (Sanai et al., 2011). This time line parallels sensitive periods during development (Kuhl et al., 1992; Benoit et al., 1996; Lewis and Maurer, 2005, 2009), which together with the recent animal observations of migration of new-born neurons from the SVZ into numerous forebrain regions (De Marchis et al., 2004; Inta et al., 2008), strengthen the hypothesis that neurogenesis may have a role in these periods. For example, neuronal migration from the SVZ to the visual cortex during the first 3–6 months of life could be involved in the sensitive period that underlies the normal development of optokinetic nystagmus (the series of reflexive eye movements elicited by a repetitive pattern moving through the visual field) which is important for the normal development of stereoscopic vision (Lewis and Maurer, 2005). In the same manner, neuronal migration from the SVZ to the auditory cortex during the first 6 month of life could be involved in the sensitive period that underlies the phonetic perception of an acquired language (Kuhl et al., 1992).

Given these results, and the previous uncovering of adult neurogenesis, it must be recognized that we are still unsure whether widespread postnatal neurogenesis occurs in regions outside of the hippocampus and olfactory bulb. Is it possible that, as was demonstrated in animal studies, neural progenitor cells exist in other areas of the lateral ventricles and that new neurons are migrating to different cortical regions during this period? As of today this is still an open question. At face value, age dependent differences in neurogenesis are a plausible mechanism for sensitive periods and for the acquisition of cognitive tasks that require multiple regions of the cortex. Fundamentally, the data that is currently available is not sufficient to determine whether widespread neurogenesis in the neocortex underlies the differences that exist between learning in early childhood and learning in adulthood.

## Summary

The sensitive periods and robust skills development seen during infancy highlight the fact that unique brain mechanisms are at work during the first years of life. Increased synaptogenesis, dendritic arborization, and myelination relative to adulthood probably play an important role. But, another, often underappreciated potential mechanism is widespread neurogenesis in the postnatal brain. An addition of new neurons would bring with it an addition of synapses, dendritic arbors and axons, all of which may contribute to the large increases in gray and white matter that are unique to these periods. Furthermore, the enhanced plasticity and naivety offered by these new neurons could contribute to the rapid development occurring at this time.
